# Supplementary figures and images for: Optical bench simulation for intraocular lenses using field-tracing technology
Source: PLoS One. 2021 Dec 15;16(12):e0250543. doi: 10.1371/journal.pone.0250543 (PMC8673660; doi:10.1371/journal.pone.0250543)

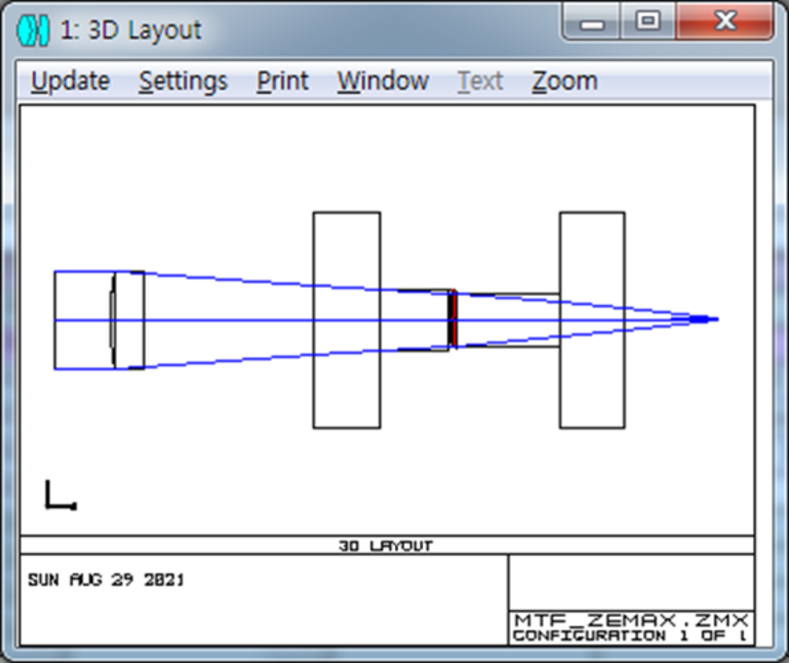

Supplement: S1 Fig — (TIF) [file pone.0250543.s001.tif]
