# Supplementary material for: Optical bench simulation for intraocular lenses using field-tracing technology
Source: PLoS One. 2021 Dec 15;16(12):e0250543. doi: 10.1371/journal.pone.0250543 (PMC8673660; doi:10.1371/journal.pone.0250543)
Supplement: Attachment — Submitted filename: Response to Reviewers.docx [file pone.0250543.s003.docx]

**Response to Editor and Reviewers for Manuscript**

Title: Optical bench simulation for intraocular lenses using field-tracing technology

### Dear Editor and Reviewers:

We express our sincere thanks for taking the time to review our manuscript and we greatly appreciate the opportunity that we have been given to further revise the manuscript. We have made the changes recommended by the editor and the reviewers.

# Reviewer 1

#### #1 The design data of IOL in ZEMAX software is missing therefore it is not possible to simulate the study and check the results.

: Thank you for your important comments. As you have pointed out, we updated the design data as supporting information just like below.

**Supporting Table 1. Optical Simulation setup**

**Supporting Figure 1. Optical Simulation setup**

#### #2 In figure 3e, There is so much difference in the strehl ratio value between ZEMAX simulation and other results. Where does this error come from?

: Thank you for your valuable comment. Fast Fourier transforms (FFTs) is a computationally efficient method of generating a Fourier transform to make the simulation of light filed. Fraunhofer, Fresnel, Rayleigh-Sommerfeld, and Kirchoff solutions are well-known formulas for calculating diffraction. But these formulas and FFTs have limitations because they are under the condition of the diffraction occurring in the no tilted screens or tilted aperture. Therefore, various formulas have been proposed to solve the error in the formulation and efficient computation of diffraction between two tilted planes. We think that the calculation error occurring in the tilted IOL plane increases as the tilted angle increases. Due to these problems, the field tracing method seems to be able to make more accurate predictions for the tilted IOL condition. We added the above to the discussion.

#### #3 In figure 4, Why do you compare 200µm decenteration with 8 degrees of tilt? 200µm in not maximum decenteration amount but 8 degrees of tilt is the maximum amount of tilt therefore I think it is not poosible to compaire the behaviour of tilt and decenteration.

: Thank you for your valuable advice. To validate our study, we refered to the study of Pieh S, Fiala W, Malz A, and Stork W.(Reference number 17) They investigated the decentration at approximately 0.15 mm intervals from 0 mm to 0.75 mm, so we also investigated the decentration at 0.1 mm intervals from 0 mm to 0.8 mm. We compared the field-tracing and ray-tracing simulation in Figure 4 and presented the values of 200 µm decentration and 8 degrees tilt as representative examples of each.

Additionally, we found and corrected small mistakes. The refractive index of acrylate is 1.47, but I wrote t incorrectly as 1.49.

Again, thank you very much for your help with our manuscript.

Best regards,

*Min Ho Kang, MD, PhD.*
