# Supplementary material for: Optical bench simulation for intraocular lenses using field-tracing technology
Source: PLoS One. 2021 Dec 15;16(12):e0250543. doi: 10.1371/journal.pone.0250543 (PMC8673660; doi:10.1371/journal.pone.0250543)
Supplement: Attachment — Submitted filename: Response to Reviewers 2.docx [file pone.0250543.s004.docx]

**Response to Editor and Reviewers for Manuscript**

Title: Optical bench simulation for intraocular lenses using field-tracing technology

### Dear Editor and Reviewers:

We sincerely thank you for taking the time to review the manuscript and thank you very much for the opportunity to further revise the manuscript. We have made changes recommended by editors and reviewers.

# Reviewer 1

#### Reviewer #1: In the ''response to reviewers'' section, Supporting table 1, the diameter of the aperture is equal to 5 mm. According to the ISO standards the IOLs are tasted with 4.5 mm and 3 mm of aperture diameter.

#### Is there any specific reason for 5 mm in diameter for pupil size?

: Thank you for your valuable comment. We conducted the study comparing the results of Pieh S, et al. (Invest Ophthalmol Vis Sci 2009;50:1264-1270.) The study of Pieh S, et al. was conducted with pupil sizes of 3 and 5 mm. So we had to choose 3 and 5 mm pupil sizes instead of the ISO standard 3 and 4.5 mm as you pointed out. In general, the smaller the pupil size, the better overall optical quality results, so we chose the pupil size of 5 mm for the discriminating power of the results. We added the above to the discussion.

Again, thank you very much for your help with our manuscript.

Best regards,

*Min Ho Kang, MD, PhD.*
